# Predictive factors related to the progression of periodontal disease in patients with early rheumatoid arthritis: a cohort study

**DOI:** 10.1186/s12903-019-0939-6

**Published:** 2019-11-08

**Authors:** Ana María Heredia-P, Gloria Inés Lafaurie, Wilson Bautista-Molano, Tamy Goretty Trujillo, Philippe Chalem-Choueka, Juan M Bello-Gualtero, Cesar Pacheco-Tena, Lorena Chila-Moreno, Consuelo Romero-Sánchez

**Affiliations:** 1 Unit of Basic Oral Investigation-UIBO, School of Dentistry, Universidad El Bosque, Bogotá, Colombia; 2 Clinical Immunology Group, Rheumatology and Immunology Department Hospital Militar Central/School of Medicine, Universidad Militar Nueva Granada, Transversal 3ª #, 49-00 Bogotá, Colombia; 3 Cellular and Molecular Immunology Group/ INMUBO, School of Dentistry, Universidad El Bosque, Cra 9 No. 131 A–02, Bogotá, Colombia; 4 Fundación Instituto de Reumatología Fernando Chalem, Bogotá, Colombia; 5 Investigación y Biomedicina de Chihuahua SC, Chihuahua, Mexico

**Keywords:** C-reactive protein, DAS28, Dickkopf-related protein 1, Early rheumatoid arthritis, Periodontitis

## Abstract

**Background:**

Rheumatoid arthritis (RA) and periodontal disease are inter-related conditions. However, factors predictive of periodontal disease progression in patients with early rheumatoid arthritis (eRA) are lacking. The aim of this study was to identify factors associated with the progression of clinical attachment loss (CAL) in interproximal dental sites of eRA patients.

**Methods:**

Twenty-eight eRA patients were evaluated for the progression of CAL at 280 interproximal dental sites at 1 year of follow-up. Markers of RA activity (rheumatoid factor, erythrocyte sedimentation rate, and C-reactive protein), a marker of bone resorption (Dickkopf-related protein 1), Disease Activity Score 28 and Simple Disease Activity Index were included as potential systemic predictive factors. Plaque index, gingival index, pocket depth, clinical attachment level and Dickkopf-related protein 1 in crevicular fluid at baseline were included as potential local predictive factors. Data were analysed in a hierarchical structure using generalised linear mixed models for progression at each site (> 2 mm) during follow-up.

**Results:**

C-reactive protein level was the most important predictive systemic factor for the progression of CAL. The mean CAL and a high degree of gingival inflammation in interproximal sites at baseline were important predictive local factors (*p* <  0.0001). Patients who received combined treatment with disease-modifying antirheumatic drugs and corticosteroids exhibited less CAL (*p* <  0.0001). The predictive value of the generalised linear mixed model for progression was 85%.

**Conclusions:**

Systemic factors, including RA disease activity and baseline periodontal condition, were associated with periodontal progression. Pharmacological treatment may affect periodontal progression in patients with early RA.

## Background

Periodontal disease and rheumatoid arthritis (RA) are chronic destructive inflammatory disorders characterised by dysregulation of the inflammatory response. The aetiology of both diseases is multifactorial, with susceptibility influenced by both genetic and environmental factors [1–3]. Periodontal disease and RA have some common aetiological and pathophysiological characteristics. Notably, both exhibit similar transition phases and stages: periodontal disease progresses from gingivitis to periodontitis [4], while RA progresses from pre-articular RA to clinical RA [5]. Similarly, the two conditions may have periods of exacerbation and remission during the evolution of the disease [6, 7].

An association between RA and periodontal disease has been reported by several studies in different populations and has been identified in a meta-analysis [8, 9]. Treatment of RA with antirheumatic drugs has been shown to reduce the progression of periodontal disease and alter clinical and microbiological periodontal variables [10, 11]; therefore, it is necessary to evaluate individuals with RA in the early stages of disease and during follow-up, in order to identify factors associated with progression of periodontal disease [12].

Although many studies have emphasised the presence of periodontitis as a risk factor for RA, very few have evaluated the reciprocal relationship. Non-surgical periodontal treatment of individuals with periodontitis and RA may lead to improvements in markers of disease activity in RA [13]; however, the presence of the RA disease could predispose patients to progression of periodontitis. Dickkopf-related protein 1 (DKK1) is a member of the Dickkopf family, an antagonist of Wnt pathway signalling, and a regulator of bone mass and joint remodelling [14]. High serum levels of DKK1 have been reported in patients with pathologies related to bone resorption, including RA and periodontitis. Low levels have been observed in patients with other conditions, including ankylosing spondylitis [14, 15]; moreover, DKK1 is a predictive biomarker for bone resorption in eRA [15, 16].

The disease activity score (DAS28) based on C-reactive protein (DAS28-CRP) is used for the assessment of disease activity in RA patients and constitutes the main parameter used to guide therapeutic decisions [17]. DAS28 is a score of disease activity score which assesses inflammation in the 28 joints most frequently involved in RA; it can be used in combination with acute phase markers, such as erythrocyte sedimentation rate (ESR) or CRP [18]. High-sensitivity CRP (hs-CRP) is a good predictor of the development of RA, in combination with metalloproteinase-3 [19]; elevated CRP levels over a prolonged period indicate higher levels of bone loss, because inflammation may contribute to the pathophysiology underlying impaired bone metabolism [20].

In 2014, Kinney et al. assessed the ability of a panel of crevicular fluid (CF) biomarkers to predict periodontal disease progression; these CF biomarkers, combined with evaluation of oral pathogens and clinical features, provide a sensitive measurement of the progression of periodontal disease. However, no studies have assessed the predictive value of other markers—used to assess the activity and bone involvement of eRA—to characterise the progression of periodontal disease [21].

The aim of this study was to evaluate the predictive value of disease activity markers in the early stages of RA, biomarkers of bone resorption, and clinical status of periodontal disease in the progression of periodontitis in a multilevel analysis using generalised linear mixed models (GLMMs).

## Methods

### Study design

This was a cohort study of patients who had been diagnosed with eRA less than 2 years of diagnosis and 1 year of follow-up was performed.

### Population

This study included patients who had recently been diagnosed with eRA (less than 2 years of diagnosis) by rheumatologists in the Department of Rheumatology and Immnunology at the Hospital Militar Central and Fundación Instituto de Reumatología Fernando Chalem, from June 2015 to February 2017. The diagnosis of eRA was based on the 2010 American College of Rheumatology and the European League against Rheumatism criteria [22].

### Inclusion and exclusion criteria

**Fig. 1:**
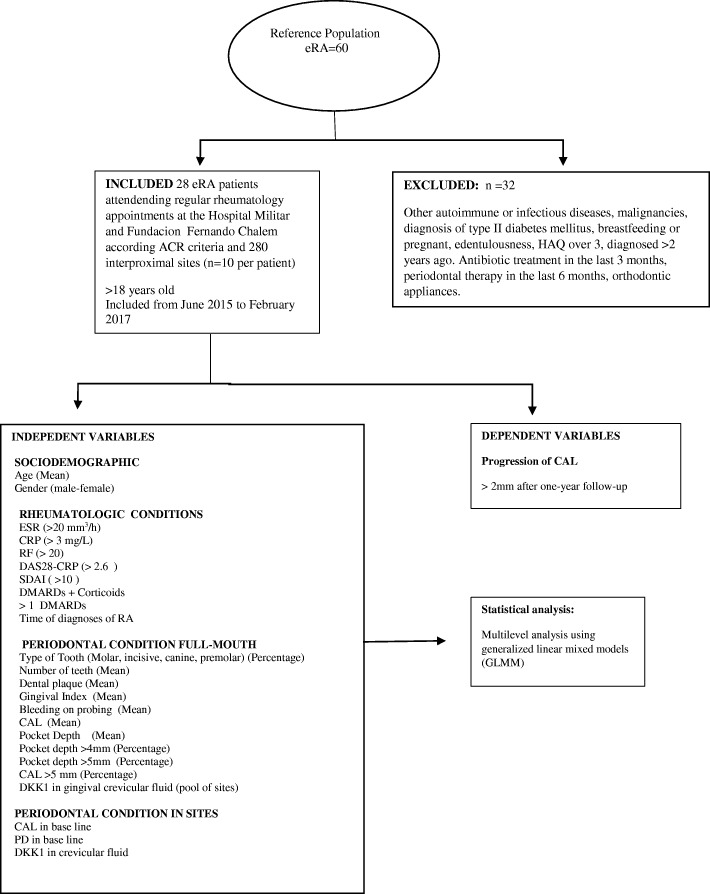
Flow chart of study protocol. ACR = American College of Rheumatology, ESR = Erythrocyte sedimentation rate, CRP = C-reactive protein, RF = Rheumatoid Factor, DAS28 = Disease Activity Score 28, SDAI = Simple Disease Activity Index, DMARDS = Disease-modifying antirheumatic drug, CAL = clinical attachment loss, PD = Pocket Depth, DKK = Dickkopf-related protein 1, HAQ = Health assessment questionnaire disability index

Twenty-eight patients with eRA were included, all of whom were >  18 years of age. Ten sites were selected in each patient based on their probabilities for clinical attachment loss (CAL) over 1 year of follow-up. The sites were selected based on the > 2 mm CAL and > 2 mm pocket depth (PD) in interproximal sites in order to include a sufficient number of sites with risk of CAL. Patients were followed for 1 year and did not receive periodontal treatment during follow-up. Individuals were excluded if they had ongoing infections, neoplasias, other autoimmune diseases, type II diabetes mellitus, or health assessment questionnaire disability index (HAQ) > 3; they were also excluded if they had undergone antibiotic treatment in the past 3 months, periodontal therapy in the last 6 months, had orthodontic appliances, or were breastfeeding or pregnant **(**Fig. 1**)**.

### Periodontal examination

All patients were evaluated by two calibrated periodontists, who performed periodontal examinations. A full-mouth examination was performed including selected sites on each permanent tooth, excluding third molars. All patients were evaluated for periodontitis based on the 2017 criteria of the World Workshop on the Classification of Periodontal and Peri-Implant Diseases and Conditions; the stages of periodontitis were determined based on CAL and PD values in interproximal sites [23]. Additionally, periodontal indices, including PD, (Inter-examiner intra-class correlation coefficient [IE-ICC], 0.94–0.96); CAL (IE-ICC, 0.92–0.96); bleeding on probing (IE-ICC, 0.88–0.90); plaque index (IE-ICC, 0.94–0.98); and gingival index (IE-ICC, 0.85–0.90), were evaluated in full-mouth examinations including the selected sites. All measurements were performed using a UNC15 probe (Hu-Friedy Mfg Co. Inc. Qulix™) at baseline and 1-year follow-up.

### CF samples

CF samples were obtained from each of the 10 selected interproximal dental sites (one per tooth) using paper strips (Perio-paper®; Oraflow, Amityville, NY, USA) that were introduced into the gingival sulcus for 30 s; cotton rolls were used to avoid contamination with saliva [24]. A Periotron 8000 (Oraflow) was used to measure the volume of CF collected (measured in Periotron units). Paper strips were removed and immediately placed in sterile tubes containing 150 μL of phosphate-buffered saline (Cat. No. 10010023; Thermo Scientific, Rockford, IL, USA) and protease inhibitor cocktail (Cat. No. 78429; Thermo Scientific). The supernatant was subsequently removed and the samples were stored at − 20 °C until evaluation.

### Joint outcome measurements

Joint activity was measured using the DAS28-CRP and the Simple Disease Activity Index. Serum samples were collected for evaluation of rheumatoid factor (RF TEST, Cat. No. 1107105; Spinreact, Santa Coloma, Spain), hs-CRP (Immulite 1000, Cat. No. LKCRP1; Siemens, Erlangen, Germany), and anticitrullinated peptide antibodies (Quanta Lite® CCP 3.1 IgG/IgA ELISA, INNOVA Diagnosis, San Diego, CA, USA). Erythrocyte sedimentation rate was measured by photometry (Test 1 THL Ali-FAX®, Polverara, Italy). As a marker of bone resorption, levels of DKK1 were evaluated in CF and serum using a sandwich ELISA (Human Dickkopf-Related Protein 1 (DKK1) Cat. No. MBS026914, MyBiosource®, San Diego, CA, USA); the detection limit was 2.0 ng/mL.

### Progressive sites in follow-up

The selected sites were evaluated at baseline and at the 1-year follow-up; they were considered progressive if they exhibited CAL of > 2 mm between the two evaluations, based on the criteria of the Consensus Report of the 5th European Workshop in Periodontology [25].

### Statistical analysis

Measures of central tendency and dispersion were determined for continuous variables, whereas frequency distributions were calculated for categorical data to identify predictor variables. The criterion for disease progression was CAL > 2 mm between baseline and the 1-year follow-up. GLMMs [26] were implemented to determine the relationship between the response variable and the corresponding predictors. In this analysis, it was assumed that the data did not follow the assumption of independence, because the evaluated sites were inside the same mouth; thus, traditional statistical methods were not adequate to describe this class of models. Conditional GLMMs are considered an important tool when describing such behaviours in data with a hierarchical nature [26, 27]. The hierarchical structures in this analysis were: 1) the first level corresponded to the site; 2) the second level corresponded to the tooth in each individual. These structures were nested and had natural heterogeneity, which includes the presence of random effects. Because the response variable (progression) was binary (i.e. belonged to a binomial distribution), it was necessary to use a canonical logarithmic link function to characterise the linear relationship of the response variable with the covariates at the individual level. However, model estimates were interpreted as log-odds ratios to ensure correct analysis. The statistical analysis was developed with R package version 3.4; the models were adjusted using the ‘lme4’ library.

The factors included in the first model are shown in Fig. 1: sociodemographic characteristics, rheumatologic condition, full-mouth periodontal condition, and site-specific periodontal condition. Smoking habits were not included because only one patient was a current smoker. The models were obtained after separately adjusting general linear models with binomial responses for each variable predictor. Successive models were developed to remove apparently unnecessary terms and achieve significant improvement until the final model was obtained. To select the best and most parsimonious model, a comparison test was performed between models using the ANOVA method, based on the lowest value of the Akaike information criterion. Results were considered statistically significant when *p* <  0.05.

## Results

### Sociodemographic characteristics and rheumatologic condition in patients with eRA

The 28 patients included in this study had an average age of 47.5 ± 12.69 years (range, 19–66 years) and 75% were women **(**Table 1**).** For markers of RA, 35.7% of patients had erythrocyte sedimentation rate levels > 20 mm^3^/h, 42.8% exhibited anticitrullinated peptide antibody levels > 20 EU, 60.7% had CRP levels ≥3 mg/L, and 67.86% of patients had a positive RF test. In relation to the measures of disease activity, 67.9% of patients had DAS28-CRP > 2.6 and 75% had a Simple Disease Activity Index score >  10. Regarding treatment, 92.9% of patients were receiving more than one disease-modifying antirheumatic drug (DMARD), and 71.4% of patients were being treated with combined DMARD and corticoid therapy **(**Table 1**).**

**Table 1:** Demographic and rheumatologic status at baseline

VARIABLE	
Age (Mean ± SD)	47.5 ± 2.6
Sex (n %)	
Male	7 (25.0)
Female	21 (75.0)
ESR (mm/h)	
Negative F (%)	18 (64.3)
Positive > 20	10 (35.7)
APCAs (EU) (n %)	
Negative	16 (57.2)
Positive > 15	12 (42.8)
CRP (mg/L) (n %)	
Negative	11 (39.3)
Positive > 3	17 (60.7)
RF (n %)	
Negative	9 (32.1)
Positive > 20	19 (67.9)
DAS28-CRP (n %)	
≤ 2.6	9 (32.1)
> 2.6	19 (67.9)
SDAI (n %)	
≤ 10	7 (25.0)
> 10	21 (75.0)
DMARDs + CORTICOIDS (n %)	
No	8 (28.6)
Yes	20 (71.4)
> 1 DMARDs (n %)	
No	2 (7.1)
Yes	26 (92.9)

*SD* Standard deviation, *ESR* Erythrocyte sedimentation rate, *APCAs* Anticitrullinated protein antibodies, *CRP* C-reactive protein, *RF* Rheumatoid factor, *DAS28* Disease Activity Score 28, *SDAI* Simple Disease Activity Index, *DMARD* Disease-modifying antirheumatic drug, *F* frequency (%) percentage

### Baseline periodontal condition in patients with eRA

Twenty-six patients (92.8%) had periodontitis at baseline; 16/28 (57.1%) had stage I, 4/28 (14.3%) had stage II, and 6/28 (21.43%) had stage III; only 2/28 (7.1%) did not have periodontitis. The periodontal indexes (full mouth, interproximal sites, and selected sites) are shown in Table 2**.** For all indexes, the interproximal sites in full mouth and selected sites examinations were similar, but were more severe than the full mouth indexes that included all sites with PD > 4 mm and CAL > 5 mm.

**Table 2:** Periodontal condition in full mouth, interproximal full mouth and selected sites

Variables	Full mouth	Interproximal	Selected sites
Number of teeth			
Number (Mean ± SD)	21 ± 7	21 ± 7	10
Plaque index			
Percentage (Mean ± SD)	61 ± 25	66 ± 27	68 ± 29
Gingival index			
Percentage (Mean ± SD)	56 ± 23	58 ± 28	50 ± 24
Bleeding on probing			
Percentage (Mean ± SD)	32 ± 28	41 ± 32	38 ± 30
Pocket depth			
Millimetres (Mean ± SD)	1.97 ± 0.3	2.74 ± 0.6	1.91 ± 1.8
Clinical Attachment level			
Millimetres (Mean ± SD)	1.55 ± 1.1	2 ± 1.4	2.35 ± 1
PD > 4 mm			
Percentage (Mean ± SD)	3.7 ± 3.2	16.7 ± 24**	18 ± 38**
PD > 5 mm			
Percentage (Mean ± SD)	4.2 ± 1.8	5.7 ± 17	8.9 ± 28
CAL > 3 mm			
Percentage (Mean ± SD)	23 ± 24	40 ± 38	35 ± 47
CAL > 5 mm			
Percentage (Mean ± SD)	7 ± 12	12.4 ± 20**	12.31 ± 22**

*SD* Standard deviation, *CAL* Clinical attachment loss, *PD* Pocket depth

Interproximal mean of percentage of interproximal sites > 3 mm in full mouth

** *p* <  0.05 significant differences relative to full mouth

### CAL in progressive sites

Among sites analysed, 15% (42/280) had CAL > 2 mm during the study period; these were classified as progressive. Twenty-four patients had progressive sites; 12/28 patients (42.8%) had between 1 and 3 progressive sites, while 12/28 patients (42.8%) had > 5 progressive sites; only 5 patients had no progressive sites. A significant correlation was observed between the number of progressive sites and the periodontal stage at baseline, as determined by Spearman rank correlation (rho = 0.42; *p* <  0.05).

### GLMMs of variables associated with CAL by bivariate analysis

GLMMs were used to analyse the numbers of sites with progression of CAL for each patient in a repeated model, and to identify systemic and local factors associated with the progression of CAL. Using CAL > 2 mm as the dependent variable, the continuous variables with the strongest associations were average CAL and average gingival index at baseline. Among the nominal variables, those associated with RA disease activity, including CRP > 3 mg/L, were predictive of a greater number of progressive sites. In contrast, the use of combined DMARD-corticosteroid treatment and the presence of bleeding on probing at baseline were predictive of fewer progressive sites. DKK1, rheumatoid factor, erythrocyte sedimentation rate, treatment with one DMARD, plaque index, PD in full mouth and periodontal condition in selected sites were not predictive factors for the presence of progressive sites. The criteria for choosing the best model were the lowest Akaike information criterion and BIC values. The p logit function was used to establish a predictive model for the response variable, CAL > 2 mm, which allowed evaluation of the predictive value. The predictive value of the model was 85%. The interpretation of the GLMM model is detailed below.

The intercept is allowed to vary randomly for each site; for a one-unit increase in age, the expected log odds of CAL > 2 mm increases by 0.008-fold. It is expected that patients with CRP levels > 3 mg/L have 1.68-fold higher log odds of sites with CAL > 2 mm (progressive sites) than patients with a lower value (< 3 mg/L). Patients treated with DMARDs-corticoids are expected to have 1.37-fold lower log odds of sites with CAL > 2 mm than patients who are not medicated with this combination. For a one-unit increase in CAL, the expected log odds of sites with CAL > 2 mm increases by 0.028-fold; for a one-unit increase in bleeding, the expected log odds of CAL > 2 mm decreases by 3.55-fold **(**Table 3**).** All estimates are in log-odds units, but were converted back to probabilities to evaluate the predictive value. The predictive factors in the model were older age, higher CRP level, combined DMARDs-corticoids therapy, greater mean CAL at baseline, and fewer sites with bleeding, which predicted approximately 85% of the risk of periodontitis progression.

**Table 3:** Generalised linear mixed model for clinical attachment loss > 2 mm

		CAL > 2 mm		
	Estimated	Standard error	Z value	*p value*
Age	0.08	0.02	3.16	< 0.0001
hs-CRP	1.68	0.54	3.07	< 0.0001
DMARDs-Corticoids	−1.37	0.52	−2.60	< 0.0001
CAL	0.02	0.01	2.58	< 0.0001
Gingival Index	2.66	0.97	2.69	< 0.0001
Bleeding	−3.55	1.02	−3.47	< 0.0001

*hs-CRP* high-sensitivity C-reactive protein, *DMARD*, Disease-modifying antirheumatic drug, *CAL* Average initial clinical attachment loss in in interproximal sites at baseline; Gingival Index, average of gingival index in interproximal sites at baseline; Bleeding, percentage of surfaces with bleeding on probing in interproximal sites at baseline

## Discussion

RA and periodontal disease are chronic conditions characterised by inflammation and involvement of bone tissue. The association between periodontal infection and the risk of developing RA has been evaluated by clinical epidemiology and basic science studies in recent years [28]; however, the progression of periodontal disease in patients with eRA has not previously been investigated and very few studies have evaluated both pathways in the manner of the present study.

The systemic condition of the patients included in this study may have predisposed them to periodontitis progression, as our results demonstrated that factors such as the activity of eRA (assessed by hs-CRP) were associated with CAL progression. Patients with RA and periodontitis presented with significantly higher clinical activity index scores in previous cross-sectional studies [12, 29, 30]. hs-CRP is used as a biomarker of inflammation associated with RA, and is reportedly elevated in patients with periodontitis [30–32]. CRP is primarily produced in the liver in response to inflammatory cytokines, such as IL-6, IL-1, and TNF-α; however, extra-hepatic production in the cardiovascular system has also been reported [32]. In 2013, Kalra et al. [33] studied the correlation between CRP in serum and in CF in patients with chronic periodontitis, with and without diabetes mellitus, compared with healthy individuals; they reported that CRP levels were increased in patients who had both periodontitis and diabetes mellitus, suggesting that the presence of a systemic condition can affect the levels of inflammation mediators, both locally and systemically. In 2010, Megson et al. [34] evaluated whether CRP was produced locally in the gingival tissue, based on the hypothesis that CRP is not strictly produced by the liver, and concluded that CRP has a systemic origin in CF. Hence, CRP in CF could be indicative of systemic inflammation, rather than strictly local inflammation. In the present study, a strong correlation was identified between CRP and progression at sites with PD, supporting a localised effect of systemic inflammation in eRA patients.

Markers of bone resorption in RA, such as DKK1, were also evaluated in this study. DKK1 plays an important role in bone remodelling; serum and synovial fluid levels of DKK1 have been associated with bone erosion in animal models of RA [35]. In 2016, Seror et al. [16] reported an association of serum DKK1 with rheumatologic variables in a multicentre cohort study of 813 patients who had eRA with higher disease activity, suggesting that DKK1 may be a structural biomarker in eRA. High levels of DKK1 in CF were also associated with eRA activity in the present study, whereas high concentrations of DKK1 in serum were not associated with the progression of PD. These findings are consistent with those reported by Napimoga et al. [36], who studied the roles of SOST, TNF-α, and DKK1 in patients with chronic periodontitis and in healthy individuals; they observed that, in the periodontitis group, the levels of SOST, DKK1, and TNF-α were significantly elevated in gingival tissue, but not in serum. It is possible that DKK1 in CF may also be a marker of bone resorption in eRA. The association of CF DKK1 with eRA disease activity in prior studies warrants a need for further study in the future as a potential biomarker of joint involvement in eRA with periodontitis; notably, CF contains significant levels of components derived from the systemic circulation, and could reflect the activation of both local and systemic pathways [37]. However, the results of this study do not suggest that the levels of DKK1 in CF or serum are good predictors of CAL in patients who have eRA and periodontitis.

In past studies, it was established that early treatment of RA with DMARDs offers valuable clinical and/or radiographic benefits over several years [38, 39]. Radiographic evidence indicated that DMARDs could initially protect against bone damage and could attenuate periodontitis [16, 40]. These data are consistent with our findings that patients taking combined DMARD-corticosteroid treatment had fewer progressive sites. It is possible that the combination of DMARDs with corticosteroids potentiates anti-inflammatory effects on the periodontium, which was reflected by reduction of CAL in these patients [41]. In contrast, some authors reported that patients taking combined DMARD therapy (methotrexate and leflunomide) showed higher levels of CAL [11]; it should be noted that those patients had established RA (more than 2 years since diagnosis) and the progression of periodontal disease was not evaluated in that study. Studies in patients with chronic periodontitis followed for a similar period of time have demonstrated CAL at between 5 and 10% of sites [42]. In our patients with eRA who were controlled with DMARDs, CAL was present at 6% of sites.

The progression of CAL in eRA was also associated with the local status of periodontitis. The average CAL and average gingival index were the most important predictive factors for site progression during follow-up. These factors were also reported as predictors of progression in patients who had chronic periodontitis without systemic disease [43, 44]. However, bleeding on probing showed a negative correlation with CAL. We hypothesise that, in patients taking DMARDs, bleeding might not be an appropriate predictor or clinical marker for the progression of periodontal disease. When comparing healthy adults without periodontitis and RA patients, the patients with RA had significantly higher levels of bleeding on probing; however, those differences were absent when patients with RA had periodontitis [10]. Significantly higher plasminogen activator levels and increased plasminogen activator inhibitor-2 levels have been observed in the CF of patients with RA, possibly as a result of their systemic condition [45, 46]. It is therefore necessary and relevant to perform a more detailed study of the factors associated with increased gingival bleeding in patients with RA.

Lang and Tonetti suggested a need for multilevel risk assessment at the patient, tooth, and tooth site levels to improve predictive values of progression [46]. In this study, the use of GLMMs allowed us to characterise a hierarchical structure, generated by including different sites in each patient [26, 27]. The models incorporated systemic variables and periodontal statuses of interproximal sites of full mouth examinations and selected sites examinations; they achieved a predictive value of approximately 85%, demonstrating that evaluation of the interactions between local and systemic factors is necessary for effective prediction of CAL in these patients. However, the periodontal condition of selected sites was not a predictive factor for progression and the periodontal status of the full mouth was a more important local predictive factor. Furthermore, the number and type of teeth were included in the models, but these variables did not influence the CAL observed during follow-up.

To the best of our knowledge, there is no research in eRA patients describing potential factors associated with progression to periodontitis. These data provide strong evidence that such factors may contribute to the early stages of periodontal disease progression. eRA could represent an important therapeutic window during which intervention could dramatically modulate the development of periodontal disease.

The main limitation of this study was its relatively small number of patients, due to the difficulty in recruiting patients with eRA in relation to delayed consultations or limitations of the health system for timely referral to the specialist, as well as the large number of exclusion criteria used in this study that affected the model. Another limitation in this study was that it only included assessment of a limited number of sites in each patient because of the difficulty of collecting CF samples at all sites. It is important to continue analysing eRA patients to determine the nature of the disease association with periodontitis progression.

## Conclusions

Factors associated with eRA disease activity, including elevated levels of CRP, could be considered important predictors of CAL, in combination with local factors such as baseline CAL and high gingival index levels. Combination treatment with DMARDs and corticosteroids reduces CAL in patients with eRA. We presume that the presence of eRA may be a potential factor associated with progression to periodontitis.

## Data Availability

The datasets used and analysed during the current study are available from the corresponding author on reasonable request.

## Abbreviations

CAL: Clinical attachment loss
CF: Crevicular fluid
CRP: C-reactive protein
DAS28: Disease Activity Score
DKK1: Dickkopf-related protein 1
DMARD: Disease-modifying antirheumatic drug
eRA: Early rheumatoid arthritis
ESR: Erythrocyte sedimentation rate
GLMM: Generalised linear mixed model
hs-CRP: High-sensitivity C-reactive protein
IE-ICC: Inter-examiner intra-class correlation coefficient
PD: Periodontal disease
PD: Pocket depth
RA: Rheumatoid arthritis
